# *QuickStats:* Percentage* of Children^†^ Aged <18 Years Who Received a Well-Child Checkup in the Past 12 Months,^§^ by Age Group and Year — National Health Interview Survey, United States, 2008 and 2018^¶^

**DOI:** 10.15585/mmwr.mm6908a5

**Published:** 2020-02-28

**Figure:**
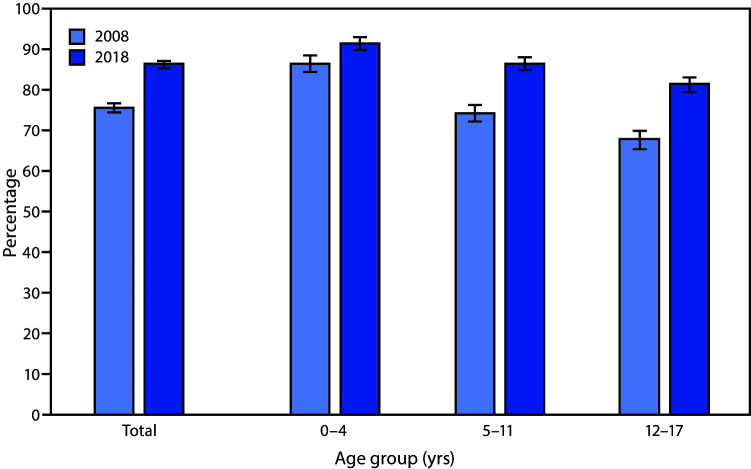
The percentage of children aged 0–17 years who received a well-child checkup increased from 75.8% in 2008 to 86.5% in 2018. Receipt of a well-child checkup increased for all age groups: from 86.7% to 91.9% among those aged 0–4 years, from 74.5% to 86.9% among those aged 5–11 years, and from 68.0% to 81.7% among those aged 12–17 years. For both 2008 and 2018, the percentage of children who received a well-child checkup decreased as age increased.

Source: National Health Interview Survey, 2008 and 2018 data. https://www.cdc.gov/nchs/nhis.htm.

